# Palliative and end-of-life care for patients with pleural mesothelioma: A cohort study

**DOI:** 10.1177/02692163241302454

**Published:** 2024-12-17

**Authors:** Donna Wakefield, Tom Ward, Hannah Edge, Catriona R Mayland, Clare Gardiner

**Affiliations:** 1 North Tees & Hartlepool NHS Foundation Trust, Stockton-On-Tees, Stockton, UK; 2 Population Health Sciences Institute, Faculty of Medical Sciences, Newcastle University, UK; 3 South Tees Hospitals NHS Foundation Trust, Middlesbrough, UK; 4 Teesside Hospice, Middlesbrough, UK; 5 The University of Sheffield, Sheffield, UK

**Keywords:** Mesothelioma, pleural neoplasms, palliative care, inequalities, socio-economic position, advance care planning, delivery of healthcare, coroner

## Abstract

**Background:**

Pleural mesothelioma is a rare and incurable cancer, with complex physical and psychological symptoms. Despite recent advances in treatment, prognosis remains poor (average 8–15 months) with a lack of research on palliative and end-of-life care.

**Aim:**

To examine markers suggestive of quality palliative and end-of-life care, including receipt of specialist palliative care, advance care planning, fewer unplanned hospital admissions at end-of-life. To compare variables with socio-economic position to identify if inequalities exist.

**Design:**

A cohort study, retrospectively reviewing the medical notes from diagnosis to death for all patients diagnosed with pleural mesothelioma between 01/01/2016 and 31/12/2021.

**Setting/participants:**

Over 5 years, *n* = 181 patients were diagnosed with pleural mesothelioma across Teesside (north-east England), *n* = 30 were alive at study commencement and excluded. For the 151-patient cohort, demographics were as follows: 92% male, 79% aged 70–89 years and 26% in the lowest socio-economic quintile (based on area-level deprivation).

**Results:**

Median survival was 246 days. Within the final 90 days of life, 69% of patients had at least 1 unplanned hospital admission, with 20% having 3+ (range 0–7). Those with the highest socio-economic position had less admissions on average. Specialist palliative care was received by patients, at home 34%, in hospital 26%, in hospice 11%. Do Not Attempt Cardiopulmonary Resuscitation (DNACPR) decisions, were in the final 24 h of life for 18% of patients (median 7 days). Disease specific findings included police attendance for expected deaths and lack of signposting.

**Conclusion:**

Patients with pleural mesothelioma have unplanned admissions to hospital towards the end of life, with possible inequalities; they receive late advance care planning and face challenges unique to their disease. It is important that patients receive high quality palliative end-of-life care through accessing specialist palliative care or have guidance/signposting to other potential sources of support.

**What is already known about the topic?**

- Pleural mesothelioma is an aggressive, incurable cancer associated with a high symptom burden, with complex physical and psychological symptoms including breathlessness, intractable pain and anxiety.
- Current research is primarily aimed at anti-cancer treatment, with an evidence gap in identifying how, when and by who palliative and end-of-life care is delivered.

**What this paper adds?**

- This study examines multiple markers suggestive of better palliative and end-of-life care for a cohort of patients with pleural mesothelioma, regardless of prognosis and performance status.
- Despite having an incurable diagnosis with a short prognosis, patients still had unplanned admissions to hospital towards the end of life. There may be inequalities, with those in less deprived area having fewer unplanned admissions.
- Around a third of patients were seen by specialist palliative care at home and around a quarter whilst in hospital. There was no evidence of signposting to an external regional specialist mesothelioma nurse; this is in contrast to previous studies where this was considered part of routine care.
- Average prognosis was only 246 days, yet do not attempt cardiopulmonary resuscitation (DNACPR) decisions were most commonly made within the final week of life, with many (18%) within the 24 h prior to death.

**Implications for practice, theory or policy**

- Whilst not all unplanned hospital admissions are inappropriate, patients with mesothelioma may benefit from earlier discussions about their wishes for the future, this may include DNACPR decisions and advance care planning to decide whether and how to potentially avoid admissions to hospital.
- Whilst not every patient requires specialist palliative care support, this should be considered for patients with complex symptoms. With documentation whether other sources of support such as a mesothelioma specialist nurse have been signposted to.
- This study was conducted in an area of high socio-economic deprivation, known to have the highest morality from mesothelioma in the country, yet inequalities exist in accessing specialist services (such as a pain service offering cordotomy) or clear guidance about what to expect after death with regard to the coroner. Services and policy should be accessible to the populations which need them most.

## Introduction

Mesothelioma is a malignancy arising from mesothelial cells,^1^ most commonly (>80% of cases) within the pleura which line the lungs. Although mesothelioma is a rare cancer with an estimated global incidence of 30,870 cases per year^2^ it is incurable and aggressive with an average prognosis of only 8–15 months. A recent systematic review identified that patients with mesothelioma suffer from a high symptom burden whilst living with uncertainty and often poorly co-ordinated care.^3^

Pleural mesothelioma is associated with complex physical and psychological symptoms^4^; which may be worse than those experiencing lung cancer but less likely to be recognised as healthcare professionals care for fewer patients with this illness. Previous studies have estimated that 90% of patients present with breathlessness and/or pain, and 90% of patients report 3 or more symptoms, including cough and fatigue.^5^ Patients are more likely to suffer from intractable progressive pain and recurrent pleural effusions than those with lung cancer.^6^ The pathophysiology of the pain, makes it extremely challenging to treat, often requiring multiple different types of analgesia.^7^ Psychological distress can also be more severe than other types of cancer, with persistent symptoms and lack of hope due to the knowledge that the disease in incurable.^8^ There is also a high caregiver burden.^9^

Approximately 85% of mesothelioma cases are related to asbestos exposure, often from industry such as shipbuilding, mining and construction.^10^ The occupational nature of the disease can add to psychological distress; with a sense of blame if exposed to asbestos during employment.^11^ Additional burdens include the complex process of seeking compensation^12^ and need for a coroner’s inquest (in England).

Evidence suggests the most people would choose to avoid hospital admissions towards the end of life.^13,14^ Therefore, fewer unplanned hospital admissions and death at home are often used as surrogate markers of better end-of-life care. Receiving palliative care has been shown not only to improve symptoms but to reduce hospital admissions and increase the likelihood of death at home. In other lung cancer, such as non-small cell lung cancer, *early* introduction of palliative care has been shown to improve quality of life and reduce aggressive medical care at end of life.^15^ However, this has not been replicated for patients with mesothelioma, where routine referral to specialist palliative care at diagnosis did not improve quality of life, and so the optimum timing of referral to palliative care remains uncertain. However, the RESPECT-Meso trial^16^ had multiple limitations, such as only including patients with a high-performance status (ECOG 0–1) and excluded those receiving chemotherapy. Additionally, those in the ‘routine care’ group, had access to a specialist mesothelioma nurse, who may play a role in meeting patient’s palliative care needs, reducing the need for specialist palliative care input. There is, however, recognised inequity in access to specialist mesothelioma nurses.^17^ There is a gap in the evidence on end-of-life care for patients with pleural mesothelioma in the real world, beyond the strict inclusion/exclusion of a trial.

Previous studies have identified inequities in palliative and end-of-life care, with those of lower socio-economic position more likely to be admitted to hospital towards end of life^18^ and less likely to access palliative care, hospice care or die at home. It is not known if inequalities in end-of-life care due to socio-economic position occur specifically for those with mesothelioma.

## Method

### Aims

1. To examine markers suggestive of high-quality end-of-life care, including fewer* unplanned hospital admissions towards the end of life, receipt of specialist palliative care, timely advance care planning and death at home, for patients with pleural mesothelioma (regardless of performance status and treatment).
2. To compare these variables suggestive of better end-of-life care with socio-economic position to identify if inequalities exist.
3. To examine other mesothelioma specific factors which may potentially contribute to better care.

(*National government statistics^19^ consider 3+ unplanned admissions as ‘frequent hospital use’, therefore 3 or more admissions will be considered for this cohort.)

Definitions and key concepts to assist in understanding the study are outlined in Box 1.

**Box 1.** Definitions and key concepts definitions/concepts.

Definitions/concepts
• Specialist Palliative Care: In the UK, specialist palliative care is free for patients to access and can be provided in hospital, at home or within an inpatient facility (e.g. hospice).
• Hospice: This provides holistic care for people with life-limiting conditions and may include symptom control, respite or end-of-life care. In the UK, a hospice is a building with inpatient beds (and often additional outpatient and day care services) where palliative care is delivered that cannot be managed adequately in other settings. (Some services within the UK also offer holistic care through ‘hospice at home’, but for the purpose of this paper, hospice refers to the specialist *inpatient* facility).
• Coroner: A coroner is an independent official who investigates deaths reported to them and makes inquiries to the cause of death, which may include ordering a post-mortem examination of the body after death or holding an inquest. Sudden, violent or unusual deaths will be referred to the coroner, in addition deaths associated with industrial disease including mesothelioma must be reported to the coroner. There are some regional variations in practice as individual coroners decide what action to take. For deaths which occur outside of normal working hours (9 am–5 pm Monday–Friday), the police may act as the coroner’s representative in some areas.

### Design

We conducted a cohort study, retrospectively reviewing routinely collected data, present in the medical notes of all patients diagnosed with pleural mesothelioma over a 5-year period across Teesside in north-east England.

### Setting

In this area, there are two National Health Service (NHS) hospitals where patients may receive their diagnosis and care. North Tees Hospital (serving a population of around 400,000 people, with 563 inpatient hospital beds) and South Tees Hospital (serving a population of 1.5 million people, with 994 inpatient hospital beds). North-east England has the highest mesothelioma rates in the UK.^20^

### Population

The inclusion and exclusion criteria for the study are outlined in Table 1.

**Table 1.** Inclusion/exclusion criteria.

Inclusion criteria	Exclusion criteria
Every adult (aged 18 years or over) Diagnosed with pleural mesothelioma between 01/01/2016 and 31/12/2021 at either North Tees or South Tees Hospital^a^ Not limited by performance status or treatment	Patients still alive at the start of data collection (11/05/2022) Patients incorrectly coded as pleural mesothelioma (such as those with peritoneal mesothelioma)

a Tissue biopsy remains the gold standard to make a diagnosis usually Computerised Tomography (CT)-guided or by Video Assisted Thoracoscopic Surgery (VATS). Patients unable to undergo tissue sampling (for example due to frailty) may have diagnosis made by multi-disciplinary team (MDT)-consensus (based on radiological, cytology and clinical information). All those given a diagnosis of mesothelioma are added to a cancer registry with ICD10 code C45

### Cohort identification

A cancer team administrator searched the cancer register for all patients diagnosed with mesothelioma (ICD10 code 45) at North Tees Hospital. This list was supplied to DW who searched the digital medical records, confirming the code was correct (removing any errors or non-pleural cases). It was important to include the entire period from diagnosis to death and so any patients still alive at study commencement were excluded (11/05/2022). Ethical approval was also granted on this basis, as this did not require consent from individual patients. A cancer team administrator at South Tees Hospital replicated the process and supplied the list to TW. A list of patient NHS numbers were sent via secure password protected NHS mail account to identify any patients known to both sites so that these could be matched later. NHS numbers were also used to search, identify and retrieve the notes of patients known to two local hospices. Once the list of included patients was confirmed, medical notes were retrieved (paper notes at South Tees Hospital and electronic medical notes at North Tees Hospital).

### Sample size

Mesothelioma is a rare disease, and so previous studies have included relatively small numbers. Therefore, a pragmatic sampling strategy was used and we aimed to include a minimum of 100 patients in the study. If there were fewer than 100 patients diagnosed within the 5-year period, then we planned to extend the study period to 6 years

### Data collection and variables

A data proforma was developed by the study team, in consultation with two PPI members. This proforma was piloted for 5 patients, by two Palliative Medicine Consultants (DW and TW) and then refined. Data were extracted by three clinicians (DW, TW, HE) who undertook initial training, where data was extracted followed by discussion on how to maximise consistency. Data extracted is summarised in Table 2, this was found by reading hospital medical notes, electronic palliative care team records, GP records (if permission given) and where relevant hospice medical records. Each patient was allocated an ID number, the secure site file linked anonymous ID number to NHS number so patients known to both hospital and/or hospice could be later linked. Data was handwritten onto the proforma, then inputted into two Microsoft Excel spreadsheet and transferred securely to be amalgamated. The combined data was imported into Stata for pooled data analysis.

**Table 2.** List of data extracted from the hospital medical notes, specialist palliative care records, primary care notes (where permission granted) and hospice medical notes (if applicable).

	Variable
Demographics	Age at death (reported within predetermined ranges)
	Gender
	Ethnicity
	Socio economic status (area level-based on IMD LSOA^a^)
Diagnosis/disease related	Date of diagnosis of mesothelioma
	Initial presenting symptom
	Basis of diagnosis
	Histology
	Performance status at diagnosis
	Occupation
	Suspected or confirmed asbestos exposure (yes/no)
	Documentation that compensation has been raised (yes/no)
	Treatment of disease: None-best supportive care from diagnosis Chemotherapy (list which) Any radiotherapy Any surgery
Outcomes	Date of death (to calculate time from diagnosis to death)
	Place of death
	Hospital use in the final year/months of life: • Number of planned hospital admissions in the final 12 months of life • Number of unplanned hospital admissions in the final 12 months of life • Number of planned hospital admissions in the final 3 months of life Number of planned hospital admissions in the final 3 months of life
	Evidence that person is known to lung specialist nurse
	Evidence person is that known to mesothelioma specialist nurse
	Evidence that person is known to hospital Specialist Palliative Care team yes/no Who made this referral and reason (if known)
	Evidence that person nis known to community Specialist Palliative Care team yes/no Who made this referral and reason (if known)
	Evidence of any other healthcare professional supporting symptom management yes/no If yes, who (e.g. cardiothoracic nurse)
	Documented that the patient had a DNACPR Yes/no/not known Date complete Role of health care professional completing DNACPR Any other evidence of advance care planning within records (e.g. Emergency Health Care Plan, Advance Decision to Refuse Treatment etc)
	Evidence of hospice admission (yes/no) • If yes, reason for referral (if known) • Any specialist interventions? (Methadone trial Y/N, ketamine trial Y/N, Nerve block referral Y/N, Cordotomy Y/N, other-please state)
	Other points of interest specific to supporting patients and families with *pleural mesothelioma*, noted when reading through the medical records (both positive and negative aspects of care)-Free text box to make notes

a Index of Multiple Deprivation (IMD) is a measure of relative deprivation in England. The IMD takes into account living conditions, including income, employment, education, crime rate and is calculated for every neighbourhood of around 1,500 people (Lower-layer Super Output Area (LSOA)). All LSOAs are ranked, and then can be broken down into deciles to make comparisons of relative deprivation between small areas. This is a place-based measure of relative deprivation based on a person’s postcode and will not apply to every person living in an area.).

### Data analysis

Statistical software (Stata/SE v.18.0) was used to tabulate the data and summarise each variable. Data was edited to enable further analysis within Stata (e.g. place of death changed format from string variable to data variable and deprivation index re-coded into quintiles). Data was displayed graphically to understand distribution.

Descriptive statistics such as mean, standard deviation and range were calculated for the variables.

Variables were compared with area-level deprivation. The primary outcome variable (number of unplanned hospital admissions in the final 30 days of life and 12 months of life) are continuous variables and the data is not normally distributed, so the Kruskall-Wallis test was used to test for a relationship. To examine the more granular detail between each SES quintile and number of unplanned hospital admissions Poisson regression was also conducted. To test for variation between SES quintile and categorical variables (for example whether seen by specialist palliative care or place of death) then Chi-square test was conducted.

### Ethics

Ethical approval granted: Proportionate Review Sub-committee of the West Midlands – Edgbaston Research Ethics Committee reviewed the above application on 19 January 2022 (Reference 22/WM/0033). Sponsor: North Tees & Hartlepool NHS Foundation Trust. Organisational agreement was gained to share data that was fully anonymised between the two NHS Trusts and two hospices via encrypted e-mail (NHS mail) in a password protected file.

## Results

### Diagnosis and treatment

**Figure 1.**
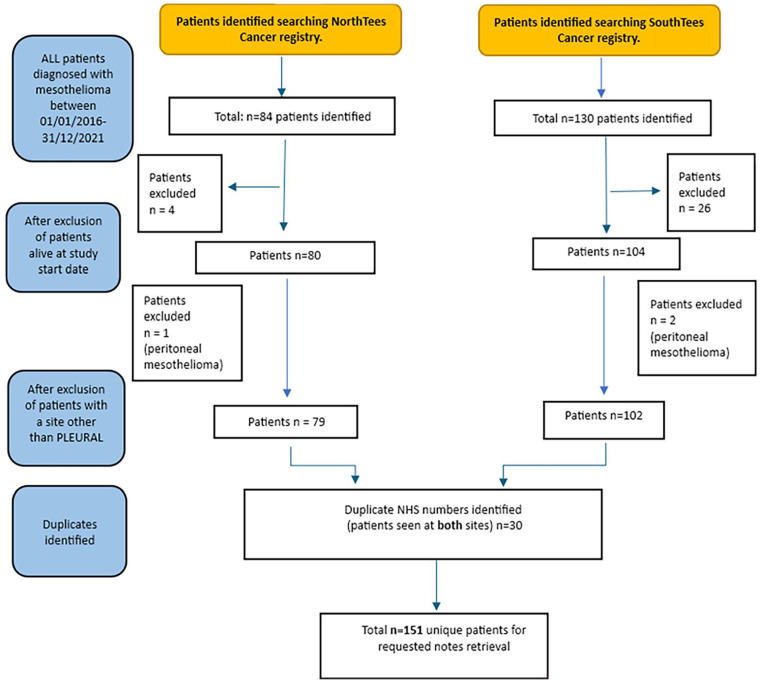
Flow diagram of patients included.

Between 1st January 2016 and 31st December 2021, a total of 181 patients were diagnosed with pleural mesothelioma. At study commencement *n* = 30 patients were still alive and so excluded, as per figure 1. Patients were predominantly male (92%) and aged between 70 and 89 years (79%). Further demographic data, diagnosis and treatment are summarised in Table 3. Shortness of breath (34%) and chest pain (19%) were the two most common symptoms at diagnosis. *Median* time from diagnosis to death was 246 days (8 months), range 0–1520 days.

**Table 3.** Demographic data, diagnosis and management of patients with pleural mesothelioma.

	Number of patients (total *n* = 151)	Percentage
Male	139	92
Female	12	8
Age range (years) (at time of death)	*N*	%
<50	1	1
50–59	1	1
60–69	22	15
70–79	61	40
80–89	59	39
90+	7	4
Socio economic status (SES)^a^	*n*	%
1 (most deprived area)	22	15
2	17	11
3	13	9
4	13	9
5	14	9
6	14	9
7	9	6
8	21	14
9	21	14
10 (least deprived area)	7	4
ECOG	n	%
0	38	25
1	43	28
2	25	17
3	21	14
4	7	5
Unknown	17	11
Basis of diagnosis	*n*	%
Histology – VATs	75	50
Histology – CT guided biopsy	34	23
Histology – U/S guided biopsy	2	1
Histology – EBUS	6	4
Cytology – pleural aspirate	6	4
Radiological only	25	16
Unknown	3	2
Histology	*n*	%
Epithelioid	62	41
Sarcomatoid	17	11
Biphasic	10	7
Mesothelioma unspecified	28	19
No histology obtained	31	20
Unknown	3	2
Treatment	*n*	%
No treatment offered	54	36
Patient declined any treatment	8	5
Chemotherapy	63	42
Including		
• Carboplatin and pemtrexed	51	34
• Cisplatin and pemtrexed	4	3
• Chemotherapy-not specified	8	5
Radiotherapy for pain	16	10
Immunotherapy/targeted therapy including:	13	9
• Nivolumab/ipilimumab (x2 privately funded)	11	7
• Bevacizumab (private)	1	<1
• Pembrolizumab (private)	1	<1
Surgery (as part of MARS2 trial)	4	2
Died before treatment decision	2	1
Not known	14	9

Total *n* = 151. Ethnicity was recorded inconsistently, so is not reported here.

a SES: based on the Index of Multiple Deprivation (IMD) ranking (a relative measure of deprivation based on postcode in England).

### Unplanned hospital admissions towards the end-of-life and place of death

In the final 90 days of life, 69% of patients had an unplanned admission to hospital, with 20% of patients having frequent (3+) unplanned admissions (Table 4).

**Table 4.** Unplanned hospital admissions in the final 90 days and 12 months of life (green shading = no admissions, amber shading = 1-2 admissions, red shading = 3+ admissions to hospital).

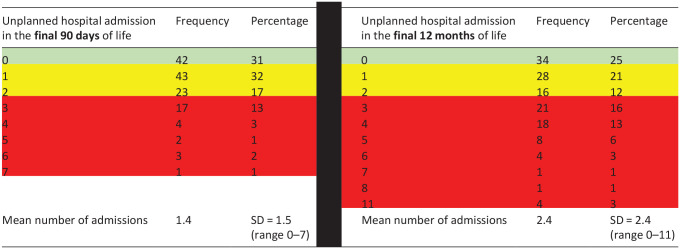

Missing data *n* = 16.

There was no statistically significant difference overall between socio-economic position and number of unplanned hospital admissions in the final 90 days of life (*p* = 0.066) or final 12 months of life (*p* = 0.174). Although Poisson regression did indicate an association between higher socio-economic position (in quintiles 3, 4 and 5) and lower number of unplanned hospital admissions, as seen in table 5.

**Table 5.** Relationship between socio-economic position and number of unplanned hospital admissions in the final 90 days and 12 months of life.

Area-level deprivation	No of patients	Mean hospital admissions in final **90** **days** of life	Standard deviation	Poisson regression				
				Coefficient	Standard error	*p* value	95% confidence interval	
Quintile 1 (most deprived)	36	2.056	1.820	–	–	–	–	–
Quintile 2	22	1.429	1.248	−0.363	0.216	0.093	−0.788	0.060
Quintile 3	26	1.231	1.177	−0.513	0.212	0.015	−0.927	−0.098
Quintile 4	28	0.892	0.956	−0.834	0.231	0.000	−1.287	−0.380
Quintile 5 (least deprived)	24	1.25	1.594	−0.497	0.216	0.022	−0.921	−0.073
Area-level deprivation	Kruskall-Wallis test *p* = 0.066			*p* = 0.00			95% confidence interval	
	No of patients	Mean hospital admissions in final **12** **months** of life	Standard deviation	Coefficient	Standard error	*p* value		
Quintile 1 (most deprived)	36	2.944	2.787	–	–	–	–	–
Quintile 2	22	2.545	2.668	−0.146	0.165	0.378	−0.469	0.178
Quintile 3	26	2.461	1.923	−0.179	0.158	0.258	−0.489	0.131
Quintile 4	28	1.785	2.331	−0.500	0.172	0.004	−0.836	−0.164
Quintile 5 (least deprived)	24	1.750	1.800	−0.520	0.182	0.004	−0.878	−0.163
	Kruskall-Wallis test *p* = 0.174					*p* = 0.00		

Place of death was identified for *n* = 101 patients (67%), of these patients’ home was the commonest place of death (50%), as seen in table 6. On Chi-square testing, there was no statistically significant difference between place of death and socio-economic position (*p* = 0.463).

**Table 6.** Place of death (if known).

Place of death	Frequency	Percentage
Own home	50	50
Hospital	34	34
Hospice	11	11
Nursing home	4	4
Residential home	1	1
Other	1	1

### Receipt of specialist palliative care

Whilst at home (own home/nursing/residential home) 34% of patients were seen by specialist palliative care. Most patients (85%) had an admission to hospital between diagnosis and death (planned and unplanned combined) and so had the opportunity to be seen by the hospital specialist palliative care team. Overall, 26% (*n* = 39) of patients received hospital specialist palliative care, as shown in table 7. Overall, 44% (*n* = 67) of patients received specialist palliative care either at home, hospital or hospice. There was no statistically significant difference between socio-economic position and receipt of palliative care (Chi-square test of association between SES and community SPC *p* = 0.498, hospital SPC *p* = 0.678, any SPC *p* = 0.876).

**Table 7.** Receipt of specialist palliative care, source of referral and reason for referral (if known).

	Frequency	Percentage	Reason for referral (if known)	Which healthcare professional made the referral to SPC (if known)
Patients seen by the **community** specialist palliative care team	51	34	• Pain (*n* = 20) • Multiple symptoms (*n* = 9) • Shortness of breath (*n* = 6) • Psychological support (*n* = 5) • Family support (*n* = 1) • Confusion (*n* = 1) • End-of-life care (*n* = 1)	• Lung clinical nurse specialist (*n* = 18) • General Practitioner (*n* = 10) • Community/District nurse (*n* = 8) • Hospital palliative care team (*n* = 7) • Hospital ward on discharge (*n* = 3) • Respiratory consultant (*n* = 2) • Hospice (*n* = 1)
Patients seen by the **hospital** specialist palliative care team	39	26	• Multiple symptoms (*n* = 8) • Psychological support (*n* = 5) • Pain (*n* = 3) • End-of-life care (*n* = 3) • Discharge planning (*n* = 3) • Advance care planning (*n* = 1)	• Hospital ward team (*n* = 39)
Inpatient **hospice**	17	11	• Complex pain (*n* = 5) • End-of-life care (*n* = 4) • Pain and psychological support (*n* = 2) • Pain and agitation (*n* = 1) • Pain and end-of-life care (*n* = 1) • Blood transfusion (*n* = 1)	• Data not available

### Advance care planning

**Figure 2.**
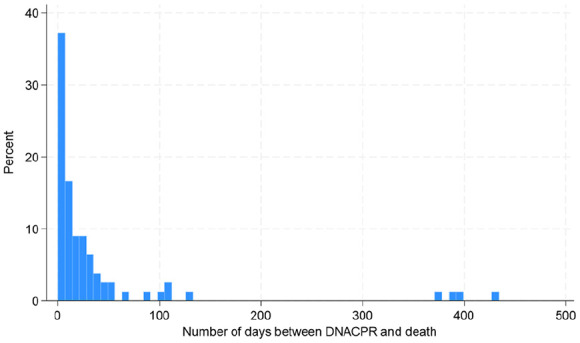
Number of days between DNACPR and death (where each bar on the x-axis represents 7 days). Data positively skewed, median time 7 days prior to death (standard deviation 88.6, range 0–431).

A DNACPR (Do Not Attempt Cardiopulmonary Resuscitation) decision was clearly documented in the medical notes for 57% (86/151) of patients. The majority of DNACPR decisions were made very late in the patient’ illness, with 18% of DNACPR discussions taking place within the 24 hours prior to death (graph 1). The most common time to have a DNACPR form completed was during a hospital admission (*n* = 34), whilst others made this decision with their General Practitioner (*n* = 25) or the Specialist Palliative Care team (*n* = 20). Further advance care planning, such as writing of an Emergency Health Care Plan (EHCP) was present in only 30% (45/151) of the patient’s medical notes (Figure 2).

### Challenges *specific* to mesothelioma

#### Mesothelioma specific support

All patients had a named lung cancer specialist nurse at the time of diagnosis. One mesothelioma specialist nurse is available across the whole north-east region, who is available via a mesothelioma support group. This is not integrated within the NHS trusts and there was no evidence within the medical notes to confirm if signposting had or had not taken place to this external support.

#### Occupational exposure/compensation

Current and previous occupation is particularly relevant in patients with mesothelioma, to identify if there could have been occupational exposure to asbestos; 70% (105/151) of patients had their occupation documented. Many patients had potential occupational exposure through working at the local chemical manufacturing plant (*n* = 17), steel works (*n* = 15), power station (*n* = 8), shipyards (*n* = 5) and coal mine (*n* = 2). Patients may wish to seek compensation from their employer/previous employers for asbestos exposure. It was documented in the patient’s records that the possibility of claiming compensation had been discussed in 28% of cases.

#### Interventional pain interventions (Cordotomy)

Whilst previous papers have highlighted the potential benefits of cordotomy to treat refractory pain in mesothelioma, no patients were referred to cordotomy due to a lack of local services.

#### Metastatic disease

Although mesothelioma is usually considered to be localised and not to metastasise, there were 14 patients who had documented metastatic disease (brain and bone *n* = 3, bone *n* = 4, peritoneal *n* = 3, liver *n* = 1, adrenal *n* = 1, widespread in multiple organs *n* = 1). This was an unexpected finding, picked up within the free text comments and so could not be quantified further.

#### Death and the coroner

As mesothelioma is an industrial disease then the coroner must be notified about all deaths. Free text comments included that for 10 patients, death at home was expected, however as the deaths occurred outside of normal working hours, the police were called to attend (as the coroner’s representative). There is a geographical variation in this practice, which would benefit from clear area-specific guidance, so that relatives are prepared for this.

## Discussion

### Main findings

This is the first study to examine the receipt of specialist palliative care and other markers suggestive of quality end-of-life care for **all** patients diagnosed with (and dying of) pleural mesothelioma over a 5-year period. There are few studies examining palliative and end-of-life care for patients with pleural mesothelioma, and what studies there are tend to exclude those with a poorer performance status.^16^

Mesothelioma is known to have a high symptom burden, which is complex to address to improve quality of life. For this cohort, specialist palliative care was received for 34% of patients whilst at home, for 26% whilst in hospital and 11% were admitted to hospice. Although not all unplanned hospital admissions are inappropriate, evidence suggests that generally people would prefer to avoid hospital admissions and spend more time at home if possible.^13^ This study found that 69% of patients with pleural mesothelioma had an unplanned hospital admission in the final 90 days of life, with some patients having as many as 7 admissions. This is higher than the national average (2021),^21^ where only 7% of patients had 3+ unplanned hospital admissions in the final 90 days of life.

Despite mesothelioma being an incurable cancer, with a very poor prognosis, discussions regarding the patient’s wishes and preferences for the future, such as Do Not Attempt Cardio-Pulmonary Resuscitation (DNACPR) decisions, are frequently left until the final days of life.

### What this study adds?

#### Potential regional inequalities

Pain was the most common reason for referral to community specialist palliative care and inpatient hospice, suggesting that management of pain is a key area where specialist expertise is valued. Pain is progressive and often complex with multifactorial aetiology^22,23^; this can make it more severe and challenging to manage than pain in lung cancer. Radiotherapy has a well-established role in pain control,^24,25^ recommended by the British Thoracic Society, yet only 10% of patients received palliative radiotherapy. Previous studies have suggested that cordotomy is safe and effective for pain control in mesothelioma, with the potential to improve quality of life,^26,27,28^ however regional inequalities in access to this service exist, both within the UK and internationally.^29^ Despite north-east England having the highest rate of pleural mesothelioma in England, there are no local cordotomy services, the closest service being 120 miles away.

Previous studies have suggested that early specialist palliative care may have less of a role for mesothelioma as their needs may be addressed by specialist mesothelioma nurses. As part of the RESPECT-meso trial,^16^ patients within the control arm were seen by a specialist mesothelioma nurse as part of usual care. Within our cohort, there was no evidence that any patients were signposted to a mesothelioma specialist nurse, via a support group. This may be because there is only one specialist nurse covering an extremely large geographical area with the closest face-to-face support group in another town, in an area with poor public transport infrastructure (although online support groups are available).

#### Potential inequalities due to socio-economic position

Previous studies, not specific to mesothelioma, have identified that patients with a lower socio-economic position have an increased odds of hospital admission towards the end of life.^18^ Within our cohort, there was a statistically significant difference between those in the higher socio-economic groups and fewer unplanned hospital admissions. No statistically significant difference was found between socio-economic position and access to palliative care. However, due to missing data, numbers are small and statistical significance should be treated with caution. A prospective study with more complete data would be beneficial to test any associations between socio-economic position and outcomes such as increased unplanned hospital admissions and access to specialist palliative care.

#### Need for mesothelioma specific support

Unique to mesothelioma is the occupational nature of the disease, leading to issues associated with claiming compensation and the need for a coroner’s inquest. Compensation can be time consuming and so occupation, previous asbestos exposure and signposting to claiming compensation should be conducted early in the patient’s diagnosis. However, these discussions were documented in only 28% of cases. Police were often called to attend after a patient’s death (as representatives of the coroner), even in cases where there was an expected death at home. In England, there is currently no unifying coroner’s policy on police attendance after death from mesothelioma. Decisions are made by individual coroners,^30^ leading to wide geographical variation in practice. Relatives should be prepared in advance, with development of regional guidance on the process after death, to avoid distress which may impact on carer bereavement.

Mesothelioma is a rare cancer, with an evidence base which has evolved over the past decade. Healthcare professionals need to be up to date with new information, to best support their patients. Mesothelioma was previously considered to be locally progressive and not lead to distant metastasise, however a number of patients developed metastases, which may not have been expected. Our evidence supports other research which has shown that although rare, mesothelioma can metastasise^31,32^ and healthcare education on new evidence such as this is important when supporting patients and preparing them for what to expect.

### Strengths and limitations

The strength of this study is that it includes data on a large number of patients (for a rare disease) and is the first to examine measures which suggest better palliative and end-of-life care from the time of diagnosis with mesothelioma until after death. By including *all* consecutive patients, we can see the characteristics of patients reflected in the real-world rather than the population in clinical trials, which are restricted to those with a good performance status. Although this study was conducted in north-east England, findings are likely to apply more widely and be generalisable for patients with mesothelioma. This study did not exclude on the basis of performance status and included consecutive patients, with demographic data similar to the real-world population in terms of age, gender, performance status for those with mesothelioma.

The main limitation of this study, as with most retrospective studies, is that it is limited to what is clearly documented and accessible. There is also lack of communication across the computer systems used for patient notes, which led to substantial missing data, meaning inferential analyses were limited. It would be beneficial to have more in depth information such as reason for hospital admission, reason for referral to specialist palliative care and how symptoms were addressed. The use of patient reported outcome measures would give insight into the potential benefits of accessing specialist palliative care but are not routine practice yet in this locality.

## Conclusion

Patients with pleural mesothelioma are in an unusual position of being diagnosed with a rare rapidly progressive, incurable cancer, associated with potentially complex physical, psychological and social needs and legal/financial implications. Prognosis is short and so timing for clear communication and future planning is critical, however it is often left until the final days of life. There are potential regional and socio-economic inequalities in aspects of care, contributing to increased number of unplanned hospital admission and access to symptom control through specialist mesothelioma nurse support and availability of pain interventions. A proportion of patients accessed specialist palliative care but it is vital to expand the knowledge-base and understand the optimal timing for referral to specialist palliative care and who would benefit most from additional support. Prospective research to identify reasons for unplanned hospital admission and to proactively identify unmet needs and benefit of specialist palliative care (for example through implementing patient-related outcome measures, such as the Integrated Palliative Care Outcome Scales (IPOS)^33^ is warranted. As this a rare disease, both patients and healthcare professionals would benefit from up-to-date educational resources and clearer policy specific to mesothelioma.
